# Parents' attitudes towards hepatitis B vaccination for their children. A survey comparing paper and web questionnaires, Sweden 2005

**DOI:** 10.1186/1471-2458-7-86

**Published:** 2007-05-21

**Authors:** Eva Dannetun, Anders Tegnell, Johan Giesecke

**Affiliations:** 1 Department of Communicable Disease Control, Landstinget i Östergötland, SE-581 91 Linköping, Sweden; 2 Communicable Disease Unit, National Board of Health and Welfare, SE-106 30 Stockholm, Sweden; 3 Department of Medical Epidemiology and Biostatistics, Karolinska Institute, SE-171 77 Stockholm, Sweden

## Abstract

**Background:**

The World Health Organisation, WHO, recommends that most countries should vaccinate all children against hepatitis B. Sweden has chosen not to do so, but the issue is reassessed regularly. The objective of this survey was to assess knowledge and attitudes towards hepatitis B vaccine for children among parents living in Sweden, and to compare distribution of responses and response rate between parents answering a postal questionnaire and those responding via the Internet.

**Methods:**

A population-based cross-sectional survey, where the sampling frame consisted of all parents to a child born 2002 living in Sweden. Two independent samples of 1001 parents in each sample were drawn. All parents were contacted by postal mail. The parents in the first sample were invited to participate by answering a paper questionnaire. The parents in the second sample were given an individual user name along with a password, and asked to log on to the Internet to answer an identical electronic questionnaire.

**Results:**

A total of 1229 questionnaires were analysed. The overall response rate for paper questionnaires was 55%, and 15% for the web version. Knowledge of the disease hepatitis B was overall high (90%). A higher degree of knowledge was seen among parents with education beyond high school (p = 0.001). This group of parents also had a higher tendency to reply via the Internet (p = 0.001). The willingness to accept hepatitis B vaccine for their child was correlated to the acceptance of the present childhood vaccination programme (p = 0.001).

**Conclusion:**

The results reveal a high level of knowledge of the disease and a positive attitude to having their children vaccinated. This study also displays that the conventional postal method of surveying still delivers a higher response rate than a web-based survey.

## Background

Immunisation is a cornerstone of preventive medicine. Childhood vaccination has been demonstrated to reduce rates of vaccine preventable diseases [1,2], leading to lower morbidity and mortality. Immunisation has largely eliminated the threat of serious infectious diseases in childhood in developed countries. However, as the incidence of preventable diseases has decreased, parents' doubts about vaccine safety have increased. This was clearly demonstrated in many European countries around the year 2000, when an unsubstantiated fear of side effects led to a sudden drop in coverage with vaccine against measles, mumps and rubella [3,4]. Diminishing vaccination coverage can lead to re-occurrence of cases of vaccine preventable diseases. Parental attitudes and acceptance are of major interest – especially when introducing new vaccines. Studies have been performed in several countries to assess parental attitude both towards already established vaccine recommendations and towards possible future vaccinations for their children. A common finding in these studies is that lack of knowledge and information is seen as one major reason for parents' choice to postpone or avoid vaccination [5-8].

In 1992 the World Health Organization, WHO, recommended the majority of its member states to include hepatitis B vaccine, HBV, in their national immunization programs – based on national assessment of incidence and prevalence. Many European countries have thus introduced HBV in their programmes, but UK, Ireland, and the Scandinavian countries have so far chosen not to, mainly due to low incidence of the disease [9].

In Sweden, recommendations from the National Board of Health and Welfare concerning immunization against HBV have focused on known risk-groups such as intravenous drug users, health care workers with frequent blood contact, and children born to mothers who are carriers of hepatitis B virus. Table 1 shows the Swedish general childhood vaccination programme. The included vaccines are offered free of charge, and participation is voluntary. A vast majority, around 98 %, of Swedish children are registered from birth at a Child Health Centre, CHC, were the recommended vaccines are administered.

**Table 1:** Vaccine programme according the recommendations from the National Board of Health and Welfare, ordinance, 1996:1 and 2005:18

	**General section**					**Selective section**		
Age	Diphteria, D Tetanus, T Pertussis, P	Polio	Hib	Measles Mumps Rubella	Responsible for vaccination	Tuberculosis	Hepatitis B	Responsible for vaccination
Newborn						Children at higher risk	Children at higher risk	Practicing doctors
3 months	I	I	I		Paediatric health-care			
5 months	II	II	II					
12 months	III	III	III					
18 months				I				
5–6 years		IV						
10 years	IV				School health-care			
12 years	II							

A variety of ways to assess parental attitudes as regards vaccinations have been used in previous studies: telephone interviews [5,10], focus groups [11,13], paper questionnaires [7,14] – all of which can be quite time consuming and costly. One way to diminish the time for data collection and registration, as well as reduce the cost could be by using the Internet, and a web designed questionnaire. In studies comparing the usage of postal to web-based questionnaires, the advantages of the web version have been demonstrated, but still the postal versions have shown higher response rates [15,16].

According to Statistics Sweden over 80 % of 16–64 year olds in Sweden have access to a computer, and a similar percentage is seen for access to the Internet (> 80%) [17,18]. Bearing this in mind, and recognizing the results from other studies that have shown equivalent data quality in electronic and postal surveys, we decided to conduct a comparison using these two methods in a population-based survey.

The aims of this study were to assess parent's knowledge of hepatitis B, and their attitudes towards hepatitis B vaccination for their children in a population-based survey, and to see how these varied by a number of demographic factors, and to compare distribution of responses and response rate between the parents answering a postal questionnaire and those answering via the Internet.

## Methods

Around 100 000 children are born each year in Sweden [19]. From the national population register, two independent samples of 1001 parents were drawn, giving a total sample size of 2002 subjects. The sampling frame consisted of all parents to a child born in 2002 who were living at the same address as the child. The name and address of the mother was drawn as the first choice. The reason for this sampling frame was that we wanted to address parents with a child who would be more than 2 years old at the beginning of 2005. These children should thus recently have received the majority of vaccines recommended in the national childhood vaccination programme. These parents represent the group for which the issue of vaccination programmes should be most current. And furthermore, our inquiry should not interfere with the programme since the next scheduled vaccination for this age cohort is not due until they reach the age of five.

All parents were contacted by mail, and informed about the investigation. The parents in the first sampling arm were invited to participate by answering an enclosed paper questionnaire. The parents in the second sample were given an individual user name along with a password, and asked to log on to the Internet to answer an identical electronic questionnaire. The questionnaire was specially designed for this investigation and comprised of 15 close-ended questions covering demographics, the child's previous vaccination status, knowledge of hepatitis B, and attitudes towards including the vaccine in the national programme, and a question if they would vaccinate their own child if offered. The question on knowledge of the disease included issues such as if the disease is vaccine preventable and if the parents knew of anyone who had received such a vaccination. There was also space at the end of the questionnaire where the respondent could expand on answers given or make comments if needed. There was no reimbursement offered for the participants. The questionnaire was pre-tested on a sample of 10 parents to ensure clarity of the questions.

**Figure 1:**
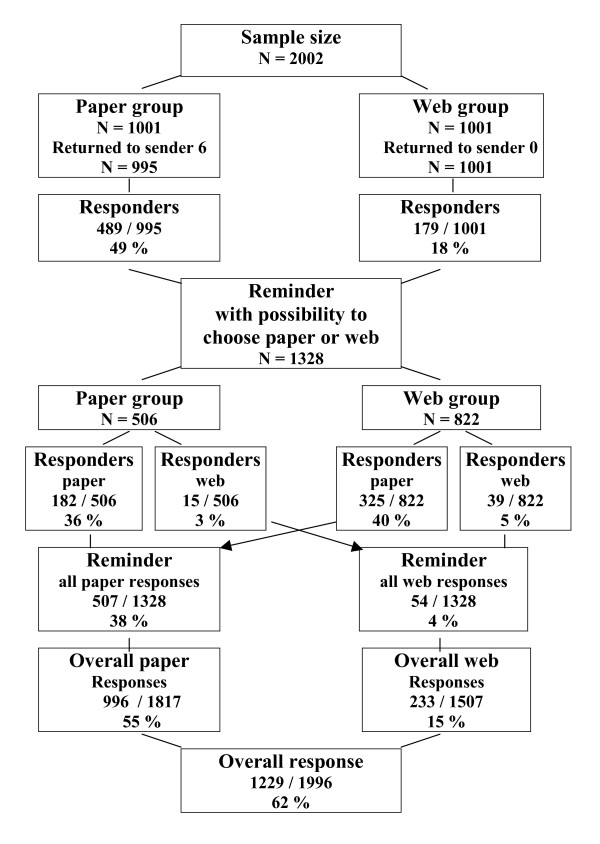
Summary of the response rate for the survey.

The investigation was performed during the months of October through December 2005. One reminder was sent out to non-responders four weeks after the additional letter. In the reminder the parents in both sampling arms were now given the option to choose between replying either by the paper or web questionnaire. The number of respondents is shown in Figure 1.

Calculations were done using JMP version 4.0.2, SAS Institute, Cory, NC, USA, and EpiInfo 6.0.4, Centers for Disease Control and Prevention, Atlanta, USA. Differences in proportions in the univariate analyses were tested by Chi square, and in the multivariate analyses logistic regression, and a level of P ≤ 0.05 accepted as significant.

The regional ethics committee of Stockholm approved the study, 2005/1028-31/1.

## Results

Two thousand and two parents were contacted by mail; of these six letters were returned to the sender because of wrong address (all from the paper-response arm). Eight questionnaires were excluded: four were incomplete and four were duplicates. A total of 1229 out of 1996 (62 %) questionnaires were completed.

### Comparison of paper versus web responses

Initial response rate was 49 % (489/995) for postal questionnaires, and 18 % (179/1001) for replies on the Internet. The reminder was sent to 1328 parents which rendered additional responses with 38 % (507/1328), and 4 % (54/1328) for postal and web questionnaires respectively. The final overall response rate for the paper questionnaire was 55 % (996/1817), and 15 % (233/1507) for the web version, see Figure 1. The majority of web responses were received within 7 days of the distribution of both the first information and the reminder: 79 % (142/179) and 74 % (40/54) respectively.

In 18 of the 996 postal replies the parent's age was missing. These data were completed with information from the national population register. There were also missing data of zip code in 20 cases in the paper questionnaires, which were completed with information from the list of addresses.

### Demographical background data

Out of the total 1229 replies, 1141 were given by the mother and 88 by the father. Median age of the respondents was 34 years, ranging from 20 to 55. Eighteen percent had one child in the family (= the one setting the sample frame), 55 % had two, 21% had three and 6 % had four children or more. Parents' level of education was; high school graduate or lower 637/1229 (52%), and education beyond high school 48 %. Fifty-four percent lived in a city with more than 50 000 inhabitants (= urban area). The majority, or 91%, lived with the child's other parent, but 2% lived with a new partner, and 7% lived as a single parent.

For the children born 2002, 98 % of the parents reported their local CHC as the prime source of information and administration of the recommended childhood vaccinations. The remaining two percent had visited a pediatrician with private practice or the pediatrics department of a hospital. Ninety-five percent of the children had been fully vaccinated. The remaining 5 % (56) had not received one or more of the vaccinations, the majority (44) not having received the vaccination for measles, mumps and rubella (MMR).

We compared the answers to the background questions between those who answered by mail and those who responded by the Internet. There were no differences in parents' age, residential area, number of children in the family, civil status, or the index child's vaccination status. The only difference between the two groups was a significantly (p = 0.001) higher tendency for parents with education beyond high school to reply via the Internet, Table 2. This was seen in both the first reply and in the overall response.

**Table 2:** Comparison of demographic data between respondents by paper or web questionnaire.

	**No. of subjects**	**No. of paper (%)**	**No. of web (%)**	**OR**	**95% CI**	**P-value**
**Parent's age**						
**≤ 34**	697	572 (82)	125 (18)			
**≥ 35**	532	424 (80)	108 (20)	1.17	0.87–1.57	0.29
**No. of children**						
**≤ 2**	906	727 (80)	179 (20)			
**≥ 3**	323	269 (83)	54 (17)	0.82	0.57 – 1.15	0.23
**Parent's education**						
**≤ High school**	637	538 (84)	99 (16)			
**> High school**	592	458 (77)	134 (23)	1.59	1.18 – 2.14	0.001
**Living in**						
**Urban area**	667	543 (81)	124 (19)			
**Non-urban area**	562	453 (81)	109 (19)	1.05	0.78 – 1.42	0.72
**Children born 2002 received all recommended vaccinations**						
**Yes**	1173	951 (81)	222 (19)			
**No**	56	45 (80)	11 (20)	1.05	0.50 – 2.14	0.89

### Knowledge and attitudes

Since no differences was seen between the mail and Internet groups, the responses to the three central questions of the study were combined in Table 3. The questions were:

**Table 3:** Responses to the three central questions of the study by the background factors assessed. All responders combined.

		**Response to "Do you have knowledge of the disease hepatitis B?"**				**Response to "Should HBV be offered to all Swedish children?"**				**Response to "Would you vaccinate your child with hepatitis B vaccine if offered?"**			
	**No. of subjects**	**No. who hade knowledge (%)**	**OR**	**95% CI**	**P-value**	**No. who should recommend (%)**	**OR**	**95% CI**	**P-value**	**No. who would vaccinate (%)**	**OR**	**95% CI**	**P-value**

**Parent's age**													
**≤ 34**	697	642 (92)				329 (47)				458 (70)			
**≥35**	532	499 (94)	0.77	0.48–1.23	0.26	248 (47)	1.02	0.81–1.29	0.83	350 (66)	1.00	0.78–1.27	0.97
**No. of children**													
**≤ 2**	906	841 (93)				420 (46)				591 (65)			
**≥ 3**	323	300 (93)	0.99	0.59–1.66	0.97	157 (49)	0.91	0.70–1.19	0.48	217 (67)	0.92	0.69–1.21	0.52
**Parent's education**													
**≤ High school**	637	573 (90)				323 (51)				424 (66)			
**> High school**	592	568 (96)	0.38	0.23–0.63	< 0.001	254 (43)	1.37	1.09–1.73	0.006	384 (65)	1.08	0.85–1.37	0.53
**Living in**													
**Urban area**	667	622 (93)				326 (49)				452 (68)			
**Non-urban areas**	562	519 (92)	1.15	0.73–1.81	0.5	251 (45)	1.18	0.94–1.49	0.14	356 (63)	1.22	0.95–1.55	0.1
**Child born 2002 received all recommended vaccinations**													
**Yes**	1173	1090 (93)				562 (48)				790 (67)			
**No**	56	51 (91)	1.29	0.44–3.48	0.8	15 (27)	2.51	1.33–4.81	0.001	18 (32)	4.35	2.38–8.05	< 0.001
**Questionnaire**													
**Paper**	996	920 (92)				459 (46)				637 (64)			
**Web**	233	221 (95)	1.52	0.79–3.0	0.18	118 (51)	0.83	0.62–1.12	0.2	171 (73)	0.64	0.46–0.89	0.006

- Do you have knowledge of the disease hepatitis B?

- Should hepatitis B vaccine be offered to all Swedish children?

- Would you have your child vaccinated with hepatitis B vaccine if offered?

In the analysis, parental factors such as age, education, number of children, etc, were regarded as the independent variables, and the population was dichotomised for each of these. For each of these variables, the responses to the three questions above were regarded as three dependent variables, and the responses in the two groups compared.

In this analysis we found that parents' age, number of children in the family or residential area of living did not affect the response to any of the central questions. Parents with a higher level of education had knowledge of hepatitis B to a significantly greater proportion, p = < 0.001. Parents with a lower level of education to a higher extent indicated that the vaccine should be offered to all children in Sweden, p = 0.006. An affirmative response to this question was also significantly influenced by the child being fully vaccinated, p = 0.001. In answer to the question "Would you vaccinate your child with hepatitis B if offered?", the factors that significantly affected a positive response were having your child fully vaccinated and replying to the questionnaire on the Internet, p = 0.001, and 0.006 respectively, see Table 3.

In the final regression model the following variables were included: age, educational level, number of children in the family, whether or not the sample child had received all the nationally recommended vaccines, and method of responding (paper/web). The logistic regression model showed the same factors to be significant as those in Tables 2 and 3.

The results of questions on details of how a vaccination programme should be organised showed that a total 66 % (808/1229) of the parents answered that they would vaccinate their child with hepatitis B vaccine if the offer was available. Of these parents 82 % (663/808) would prefer to have the vaccine offered within the ordinary schedule for childhood vaccinations, but 10 % wanted to have their child vaccinated when he/she reached a higher age. The remaining 8% (63/808) were uncertain on which alternative to prefer.

## Discussion

Our findings show that although the web-based technology is well established, the conventional postal method of surveying still delivers a higher response rate – even in a country where access to computers and the Internet is over 80 %. We also found that a high level of education clearly influenced the tendency to answer by the Internet.

If the web-questionnaire would have been administered by e-mail, this might have increased the response rate, but there is no national registry of e-mail addresses available in Sweden, which makes a population-based email survey impossible. Also in other investigations where e-mail and postal distribution have been compared, response rates were still in favour of the traditional paper version [15,16,20]. None of the web questionnaires were missing any data, since the web construction reminded the respondent if a reply was missing. The missing data in the paper questionnaires were all possible to complete, but this involved thorough confirmation and double-checking which is quite time-consuming. We have no information if the non-responders views on immunisation differ from those who responded. Although previous experience from a telephone survey among parent's who's child had not been vaccinated with MMR, was that parents with a negative or sceptical attitude towards childhood immunisations very willingly shared there opinions [5].

The question if the parents would vaccinate their child is in this kind of study on attitudes is hypothetical and the answer might not correlate to their actions if a real offer of vaccination was made. However the results gives us an indication that the attitude among most parents is positive.

The aim of this study was to assess parents' knowledge of hepatitis B, and their attitudes towards vaccinating their child with HBV. An overall knowledge of the disease was reported by 90 % of the respondents. There were no questions to verify that the respondent did not confuse their responses with knowledge of for example hepatitis A. In Denmark a study was performed 2002 assessing parents' knowledge about hepatitis B and their acceptance of immunization for their child. This investigation showed a similar high level of knowledge, 86 %, among Danish parents [21] which strengthens our result.

Knowledge of hepatitis B was highest among parents with an education post high school, while the willingness to accept HBV for their child was correlated to the demonstrated acceptance of the present program. High acceptance of the current vaccinations was also a significant factor for parents to have a positive opinion that all children in Sweden should be recommended HBV. It should be noted, however, that 48 % of parents with higher education stated that they did not have any opinion on whether such a general recommendation should be made, which shows that better knowledge of the disease does not necessarily make it easier to decide if the vaccine should be universally recommended or not (data not shown).

Previous studies have shown lack of knowledge and of information to be important factors for parents' reasons for postponing or avoiding immunizations for their children [8,22]. Since health professionals are referred to as the main source for parents' information on childhood immunization issues, the necessity for these to be up to date and well informed is of major importance [5,6,13,23]. In a study by Bardenheier et al., where they received a 52 % response rate, it was found that over 90 % of parents believed immunizations to be important for the health of their child. In this study, only a small proportion of parents had so strong concerns about vaccine safety that they had avoided having their child vaccinated with all the recommended vaccines. Furthermore, even parents whose children had received all the recommended vaccinations reported concerns about vaccine safety [24]. This result highlights the need to address concerns and to supply all parents with accurate knowledge, in an attempt to prevent them from changing their practice when facing further vaccine controversies.

Previous studies have shown that the length of the questionnaire effects the response rates and that a shorter version promotes the propensity to response [25,26]. This experience was taken into account when constructing the questionnaire for this study. The overall response rate of 62 % is still satisfactory regarding that only one reminder was sent out during a fairly short investigation period. We have no indication that lengthening the time period for data collecting would have increased the web reply rate: only 4 % (54/1328) responded by the web after the reminder, and over 70 % of the web response came within the first week of both the primary invitation and the reminder. However, an additional reminder would probably have increased the number of returned paper questionnaires.

## Conclusion

This study shows that knowledge of hepatitis B is high among Swedish parents. The high level of acceptance of the recommended childhood vaccinations seen in Sweden is perhaps the most important factor for success if HBV is introduced in the national recommendations. Awareness of parents' knowledge and opinions are of crucial importance to successfully maintain and achieve high levels of childhood vaccination, especially if new vaccines are to be introduced. Opinions and believes regarding vaccine issues tend to chance quite rapidly over time, sometimes with consequences which can effect coverage like seen with MMR [3,4]. A quick way of performing surveys to assess parents' views would be of great benefit for decision makers and primary health-care workers. Although the response rates were low by the Internet we experienced the positive effects with the web questionnaire like quick response, no risk of data entry mistakes, and facilitating analysis. Further investigations to enhance a suitable method or how to promote the usage of the Internet should be further explored.

## Competing interests

There are no conflicts of interest for any of the authors in relation to this work. The lead authors had full access to all data in the study and had final responsibility for the decision to submit for publication.

## Authors' contributions

RN PhD. Eva Dannetun had the original idea for the study and substantial contribution to the design of the paper and the acquisition, analysis and interpretation of the data. In addition Eva Dannetun was involved in drafting the manuscript and revising it for intellectual content.

MD PhD. Anders Tegnell had substantial contributions to the design of the paper and to the analysis, and interpretation of the data. In addition Anders Tegnell was involved in drafting the manuscript and revising it for intellectual content.

Prof. Johan Giesecke had substantial contributions to the design of the paper and to the analysis, and interpretation of the data. In addition Johan Gisecke was involved in drafting the manuscript and revising it for intellectual content.

## Pre-publication history

The pre-publication history for this paper can be accessed here:


